# General practitioners’ reflections on using PSA for diagnosis of prostate cancer. A qualitative study

**DOI:** 10.1080/02813432.2022.2057032

**Published:** 2022-04-12

**Authors:** Olav Thorsen, Eirik Viste, Torgeir Gilje Lid, Svein R. Kjosavik

**Affiliations:** The General Practice and Care Coordination Research Group, Stavanger University Hospital, Stavanger, Norway

**Keywords:** PSA, prostate cancer, early detection, screening, uncertainty, ambivalence

## Abstract

**Objective:**

To investigate how GPs use the PSA test as a diagnostic tool in daily practice.

**Design:**

Qualitative study using focus group interviews, the transcripts being analyzed by systemic text condensation.

**Subjects:**

A total of 17 Norwegian GPs in three CME groups.

**Main outcome measures:**

Exploring GPs’ attitudes to national guidelines and the practical use of the PSA test.

**Results:**

Detecting prostate cancer in general practice is a common and important, but difficult diagnostic issue. Our participants experienced uncertainty regarding the test when to use it, how to interpret the results and when to refer to specialist health services.

**Conclusion:**

The study revealed a general ambivalence to the use of PSA. Many patients present urological problems, and many are afraid of having cancer. PSA is commonly used, but sometimes generates problems rather than solving them.

**Implications:**

The use of the PSA test should be based on a thorough clinical assessment and in close collaboration with the patient.

**Key points**
- Many patients in general practice present urological problems, and many are afraid of having cancer.
- GPs have a general ambivalence to the use of PSA when to use it, how to interpret the results and when to refer to specialist health services.
- The use of PSA sometimes generates problems rather than solving them.

## Introduction

Worldwide, prostate cancer (PCa) causes about 359,000 deaths annually, making it the second leading cause of male cancer death [1]. Prostate Specific Antigen (PSA) is produced exclusively by the prostate gland. An elevated PSA can be related to the presence of cancer, but may also be found in other clinical conditions such as benign prostate hyperplasia (BPH), infections and inflammations of the gland.

In Norway, men presenting with urological symptoms will normally visit their regular GP for a general consultation and examination. This may include a digital rectal examination (DRE) and usually a PSA test, depending on age and heredity for PCa. When suspected of malignancy, patients are referred to a urologist for further examinations, such as MRI and biopsy. Since the PSA test was introduced in general practice in Norway in the 1990s, the testing has increased to more than 500 000 PSA analyses annually [2]. This is a paradox, considering the relatively low sensitivity and specificity of the test for cancer [3], no specific test threshold value for PCa and the fact that general screening for PCa with PSA is not recommended. In Norway, all inhabitants (5 million) have the right to have a regular GP. The mean number of patients per GP is about one thousand. The economic incentives for taking many blood tests in general practice are weak. In contrast, many private health companies recommend their customers to take a PSA test, as part of a general health check. In addition, organizations like Movember (www.movember.com) recommend men over a certain age with urological symptoms to consult their regular GP to consider having a PSA test.

Studies such as The European Randomized Study of Screening for Prostate Cancer (ERSPC) have shown reduced cancer-specific mortality, the disadvantage being many cancer cases found where treatment causes unnecessary side effects without prolonging life [4].

Norwegian guidelines recommend specific, risk-adapted screening only for men with genetic predisposition from the age of 45 years and to breast cancer susceptibility gene (BRCA) mutation carriers who have been confirmed to be at risk of early and aggressive disease (mainly BRAC2), from around 40 years of age, as recommended by the European Association of Urology (EAU) [5]. Nordic GPs are also influenced by the *Choosing Wisely Campaign* (www.choosingwisely.org), which seeks to advance a national dialogue on avoiding unnecessary medical tests, treatments and procedures. One of their recommendations relevant to primary care is: “do not recommend prostate cancer screening for men over 75 years of age without considering life expectancy and the risks of testing, overdiagnosis, and overtreatment”. Still, over-utilization of using the PSA test is quite common, often caused by patient preferences when deciding whether to order a test [6].

All authors are GP specialists and have experienced the uncertainty of using PSA in clinical practice. Research on GPs’ reflexions regarding the use of PSA is scarce [3,7–12]. In September 2017, a new blood test (Stockholm 3) for diagnosing PCa was introduced in our region [13]. Before the start of using this new test, we wanted to explore how GPs were thinking about using the PSA test, their attitude to national guidelines and the practical use of the test.

## Methods

### Design

We performed a qualitative focus group study using semi-structured interviews with 17 GPs in three established groups for continuous medical education (CME) in the Stavanger region in Norway. We applied systematic text condensation (STC), a method for thematic cross-case analysis, to analyze the focus group interviews, as described by Malterud [14].

### Participants and setting

We wanted to interview GPs with experience using the PSA test. We, therefore, recruited GP specialists, meaning that everyone had at least five years of clinical practice, which is the minimum requirement to become a GP specialist in Norway. In Norway, all GP specialists must participate in regular group meetings with up to ten participants, normally eight to ten times a year, to maintain their specialty in family medicine/general practice.

These meetings normally take place in the evening. We aimed for maximum variety in age, gender and experience. Among the around forty CME groups in our region, we chose three. We sent an e-mail to the group leaders with information about the study and asked for a focus group interview in one of their regular group meetings. No groups declined participation. The participants consisted of 17 GPs, 6 women and 11 men, age 35–67 (Tabel 1). All worked in urban practices, with a short distance to the local hospital with a urological department. The GPs gave their written consent prior to participation Table 1.

**Table 1.** Participants, gender and age.

Age groups	Female	Male	Total
30–39	1	1	2
40–49	3	2	5
50–59	2	3	5
60–69	0	5	5
Total	6	11	17

### Interviews and data collection

The focus group interviews took place in May 2017. Two of the authors (TGL and OT) participated in all group interviews and alternated as mentor and assistant. We first gave a short introduction about the study and the focus of interest: their reflections on the PSA test and practical use of the test, and their attitudes to the guidelines. The interviews lasted from one to two hours, all being audio-recorded and transcribed verbatim.

### Data coding and analysis

The interviews were analyzed using STC, a method well suited for thematic cross-case analysis [14,15]. STC consists of four steps, where the first step is to obtain an overall impression and identify preliminary themes. The second step is to develop code groups by identifying meaning units reflecting the aims of the study. Then, as the third step, we established subgroups connected to each code group, condensed the contents and identified illustrative quotations. Finally, we synthesized the condensates and recontextualised the descriptions of each category regarding different aspects of using the PSA-test and following guidelines. All authors took part in the analysis of the text. To preserve the anonymity of the informants, we chose not to mark whom the citations came from.

## Results

The participants shared their views on and experience with the PSA test, and their reflections on guidelines for diagnosing PCa. The overall impression from the interviews was the participants’ experiences with uncertainty regarding the test when to use it, how to interpret the results and when to refer to specialist health services. Many stated that their aim was to follow the national guidelines. However, many presented stories demonstrating how following the guidelines was challenging in their daily work. None of our participants used PSA as a general screening test. The participants used the test for case finding, or when urological symptoms or a genetic situation was the case or at the request of the patient. The use of information leaflets about prostate symptoms and diagnostic procedures was common.

### Professional uncertainty

All our participants had experienced situations related to PSA when they were uncertain about what to do next or what the right procedure would be for the patient. Several had experienced situations with a mismatch between a cancer patient with a low PSA and the contrary, a high PSA with no cancer found.

*I had two patients; it started a few years ago. Both of them had fairly recent prostate changes in symptoms, and thought, well… I should probably check this further, palpated (prostate), it felt abnormal, i.e. asymmetry and hard. Took PSA on both which were normal. Nevertheless, I referred both, and both had cancer.*

What the PSA threshold level should be for recommending radiology (MRI) or biopsy raised many questions and generated a lively discussion, and the participants had many questions. What is actually a normal value? And what level is normal in relation to age. When is the appropriate time to intervene, and when should the PSA test be repeated? Most participants knew the national guidelines and tried their best to follow them.

However, in practice, several had found their own pragmatic way to use the test. A participant with long experience in guiding medical students said:

*I have given up following guidelines, I have tried for many years, and it was so contradictory… Most people said ok, do not take (PSA test), do not take! I usually say to my students: I think I have done it (taken PSA test) to everyone for many years, it is a typical thing that I do in practice, which scientifically is bad practice. I take significantly more PSA than recommended.*

### The screening dilemma

Several of our participants mentioned the impact of private health companies on patients’ behavior. Many of these companies include the PSA test as one of their suggested tests in a “screening package”. Several expressed the pressure from patients to have a PSA test is influenced by social media, the Movember campaign (www.movember.com), friends and relatives. It was a difficult pressure to resist.

The clinical value of just having a PSA test result was considered low. All agreed that the national recommendation not to use PSA as a general screening test was wise. Many experienced being under a cross-pressure when patients asked for a test. Some told how they had to take a deep breath a couple of times before starting to explain the fact that this was not necessarily straightforward. It was often a difficult situation. It would be much easier if there was a better test, where the balance between benefit and cost was more advantageous.

*That’s exactly what the health authorities think is bad medical practice, that you take tests on people without them being aware of it, and when tests that are so weak, you create a problem that people do not have…. Because it pays to stick to good medical practice and it is not good medical practice to put this into a standard health screening without telling about it.*

One participant described the difficulties of meeting the fates and accusations from patients when things go wrong. They agreed upon social media presenting so much information about this test to be taken, while the PSA test as a general screening tool was considered a bad test alone. Some of the participants were men over 50, and they could relate to the patients’ need for reassurance. One of them admitted having taken PSA himself without having symptoms, just to know the value. He described himself as a patient, wanting to have the result, being happy to have a low value.

*I took it (PSA test) when I turned 55. I think it is good for me to know. A number. They can say what they want, when it comes to the individual, as our patients are as we are too, so I think the way as my patients think. Now I am a patient, not a doctor. When I then know that my PSA is 2, imagine if it was 6, or 5, at the limit, then I had to take it again in for example two years to see if it has suddenly doubled in value. A PSA value has a meaning as well.*

### The doctor-patient relationship

Many participants mentioned the long and close relationship to their patients as a reason for doing the best for them, both professionally and emotionally. They had problems saying no when a patient asked for a test, often a wish originally from his wife. Many patients came because a family member or a friend had prostate cancer. Giving the patient a “false assurance” of having a low PSA was a dilemma for many. Still, pleasing the patient by just taking a test was easier than declining to take the test and instead of explaining the uncertainty about the result. Many of the participants described the problematic situation of saying no to taking a blood test.

*In my everyday life, I think most people who ask for the test do so, not because they have a brother or a father who has prostate cancer, but because they have someone they know, who knows someone, or who has had it themselves and who has made them read a bit about this phenomenon and think that it may be an answer to the small uncertainty in relation to their health that they know about it. Moreover, my specific patient history is such a man, 47–49 years who has a colleague, or a peripheral friend who had a prostate cancer and that's why he thought he would be tested.*

Several participants mentioned the possibility to have a shared decision with the patient. The use of information leaflets was common. Our participants did not want the burden of not taking the test, risking the patient to have cancer. If things went wrong, if a patient was diagnosed with PCa after not having taken a PSA, it would be easier to accept if they had shared the decision with the patient. The GPs seldom wanted to say no when their patients had made up their minds. Many patients had already made up their minds to have a test when they arrived. The fear of being sued for malpractice was also mentioned. By sharing the uncertainty with the patient, the possibility of being seen as not following guidelines was considered lower. In practice, they informed the patients about the consequences of taking a PSA test, what further examinations were possible and relevant and what was the wisest to do.

*Then it will be as I mentioned, I say to the patient, when we take the test we can have a problem when it comes to interpretation, so you have to be aware of that. You can have cancer with a very low (PSA) value and you can have no cancer with a higher one. There are very “kind”cancers that do nothing and again there is another (worse) cancer. Moreover, the test is not good. We can take it (PSA), but it is not good.*

### A case-finding tool

Case finding is essential in general practice, as so many conditions and symptoms can mean “all-or-nothing”. Most of our participants used the PSA test as a tool in their every- day practice, as a supplement to other tests and information. For some, the “clinical view”, meaning personal clinical experience during many years was more important than guidelines.

*I used to have a clear attitude that when this topic was on the table, we talked carefully about symptoms from the urinary tract, actively tried to ask about it. Sometimes (I) got wise, but not often. Therefore, I thought it was right then to do a rectal exploration before deciding whether to do a PSA. However, the description of the rectal exploration, the findings, is not more helpful than the PSA test. So now it has also become a little more difficult I think, then there will be such a trade-off, where the patient stands, what the patient thinks, the motivation to take the test and the upper limit to interpret uncertain results.*

The use of the International Prostate Symptom Score (IPSS) was common. However, as it is possible to have a low IPSS score and at the same time have prostate cancer; it was not considered very useful. Even symptoms such as hesitation or nocturnal urination were not always symptoms that had any impact on the decision to refer. A patient’s wish for having a PSA test without any advice or physical examination was not appreciated. A more comprehensive attitude to the subject was important for many.

*A PSA test on request I think is bad practice. I say tests mean that he (the GP) has routinely asked a question, one has done a routine examination, rectal examination; all this must be part of taking a PSA test and information given to the patient at the same time. Then you have at least made sure that based on the test you take, what it means to have such a diagnosis. Just taking a test will not be ethically correct for me.*

## Discussion

To our knowledge, this is the first study on GPs’ thoughts and opinions on using the PSA test in Nordic countries. The participants discussed many aspects regarding uncertainty in relation to the test. A similar study on GPs in Australia and England analyzed the uncertainty of GPs using PSA as a diagnostic cancer screening tool [16]. They found a difference caused by English GPs having more precise guidelines and policy clarity to convey to their patients, making them less ambivalent to the practical use of the test [17].

We found that our participants had mixed feelings about the use like it is easier finding a very low or high value, indicating no or likely cancer and on the other hand the border values, creating problems concerning when to repeat the test and to refer. The thresholds for accepting PSA as normal are not universally accepted. In the USA in 1994 PSA was approved by the FDA as an aid to the early detection of prostate cancer, using a threshold of 4 ng/ml as the upper limit of normal [10]. In 1995, Loeb recommended the PSA threshold for biopsy to 2.5 ng/ml. There is, however, no threshold below which the risk for prostate cancer is zero. The determination of PSA cut-off used in clinical practice, therefore, remains controversial. Many studies have shown an uncertainty using PSA [7,18].

Hodgson *et al* (New Zealand, 2010) found that GPs had difficulty in providing patients with information about the pros and cons of PSA testing [8]. Like in Ross’ study [19], we found that belief patterns about evidence uncertainty and the efficacy of using PSA play a role in whether GPs offer PSA in a specific situation. Like in Rai’s study from 2007 (11), our participants experienced many patients to be already committed to having the test when they saw their GP. Information leaflets were used to share the responsibility for the clinical assessment with the patient. Still, when they were given the information about the benefits and limitations of the PSA test, it was often too late to alter their decision. It was easier just to take the test than to argue and risk having a dissatisfied patient.

The best available evidence from randomized trials has shown that screening has at most a small benefit in reducing prostate cancer mortality and the risk of developing the metastatic disease [20]. None of our participants used PSA as a general screening test for PCa. They were aware of the recent research, showing that screening for prostate cancer leads to a small reduction in disease-specific mortality over ten years but does not affect overall mortality, and the possible complications from biopsies and subsequent treatment [9,21]. Charvin [22] found that persons with no PCa symptoms attached greater importance to a decrease in the number of false negatives and a reduction in prostate cancer mortality than to other risks such as the number of false positives and over-diagnosis. Our participants had similar thoughts about their patients. All had experienced the demand for opportunistic prostate cancer screening, as shown in other studies [6,23].

It was very common among our participants to use PSA in their clinical work with men having urological and prostate-related symptoms, and PSA was mandatory when referring a patient to specialist services. Most of them also said that they did a digital rectal exploration (DRE) before sending a referral.

In a national system with all having a regular GP, the doctor-patient relationship is important in the way GPs manage the consultations and examinations. The importance of empathic and mutual trust is fundamental, as well as good information and shared responsibility for taking care of the actual clinical problem.

### Strengths and weaknesses

Strengths are good spread in age among the participants and good knowledge of general practice and clinical issues among the researchers. The authors who took part in the interviews as mentors and assistants (TGL and OT) are known to the participants as experienced GPs and researchers, enabling a safe atmosphere for an open discussion in the group meetings.

Weaknesses are that all the researchers were experienced general practitioners, who can contribute to blind spots and that some topics are underestimated. Among the participants, there were few very young doctors, since we only recruited specialists.

In a country with a strong primary care system based on a regular GP for all, the economic incentives for taking blood tests are weak. In our study, the doctor-patient relationship is the main reason for good medical practice, supported by national guidelines. The participants were GPs in the south-western part of Norway; thus the results reflect the conditions for GPs in the Nordic primary care system and do not necessarily explain reasons for using the PSA test in more finance-driven systems.

## Conclusions

This study revealed an ambivalence among GPs to use the PSA test. Except for patients with a familial hereditary risk of PCa, our participants were often unsure of what was best for the individual patient, i.e. if they should recommend conducting the test, how to interpret the answer, and when to refer to further examinations.
